# Electricity and Gynecology*Read before the Central Texas Medical Association, January 10, 1900.

**Published:** 1900-03

**Authors:** M. B. Grace

**Affiliations:** Seguin, Texas


For Texas Medical Journal.

### Electricity and Gynecology.*

BY M. B. GRACE, M. D., SEGUIN, TEXAS.

In presenting a paper on this subject I find myself under the embarrassing position of selecting only a few of the many diseases of the female generative organs which may be successfully treated with this important therapeutic agent. Until recently electricity was very little taught, and, in some instances, almost, if not entirely, ignored by the medical colleges. Today it is recognized as an agent of the utmost importance, especially in the branch under discussion.

*Read before the Central Texas Medical Association, January 10, 1900.

The purpose of this paper is to stimulate discussion and investigation in this direction, more than to bring out any particular line of treatment. Patterson tabulates the effects of three of the currents as follows: Primary faradism stimulates sensory and motor nerves and contracts muscles; produces slight dilatation of blood-vessels, and is slightly refreshing. Galvanism stimulates nerves and contracts muscles, drives drugs through the skin (cataphoresis), produces chemical decomposition (electrolysis), dilates capillaries strongly and refreshes nerve and muscle after fatigue. The anode allays irritation, is an analgesic, and has an hæmostatic action due to the acids collecting at the pole. The cathode is stimulating and liquefying. Both poles are caustic; the cathode more so. Nutrition is improved by the current, by the contractions of muscle, by stimulation of trophic tracts and centers, by dilatation of the blood-vessels, and by electrolysis and cataphoresis. The positive is germicidal when used in strength above fifty milliamperes. Again, we have the secondary faradic current or current of tension that is of importance in the study of this subject, the effects of which is principally sedative. This brings us to a consideration of certain pathological conditions of the uterus and appendages, only a few of which may be merely outlined in a paper of this nature. Endometritis and metritis are now generally recognized as microbic in their natures. Germ action being the original and continuing cause; resultant tissue change being the disease itself. In most cases the modes of entrance of the germs are by direct implantation through coitus, unclean instruments, etc. There are many cases, however, in which these modes of entrance can be absolutely excluded, particularly in virgins not even guilty of masturbation. The explanation is probably through the fact that the vagina and cervix as far as the internal os is normally the home of pathogenic germs. The absence of these germs from the uterine cavity we are told is due to the fact of a barrier of phagocytic sentinel cells situated in or about the internal os. As long as the general health is maintained these cells are enabled to repel the invasion from below. With the health impaired, the resisting power of these cells is weakened, hence the painful menstruation and leucorrhœa so often experienced by anæmic young girls. The cure of any form of metritis necessitates the alteration of abnormal nutritive processes.

Tissue proliferation being the condition found in all instances of thoroughly developed metritis, endometritis and endocervicitis, nature must be stimulated and assisted in the throwing off of this altered material. Where the mucous membrane alone is involved we could naturally conclude that at least a part of the curative effect of electricity would be due to its direct destruction of diseased tissue by the electrolytic or polar action of the galvanic current. We also get the effect of tissue stimulation. In using the bare metallic electrode, twenty milliamperes for five minutes is necessary to produce only a slightly cauterant effect. Again, an effect to be desired is that on the tone of the nerve filaments, the anode being sedative and the cathode stimulating. In the mere local application of solutions of bichloride, creosote, iodine, iodized phenol, etc., we get only the local cauterant and antiseptic effect; but when applied on the positive pole we have the advantage of the antiseptic effect on the deeper tissues. When I have reason to believe that the sub-mucous tissues are involved it is my habit, after using the bare positive electrode of block tin (intra-uterine) from five to eight minutes for its cauterant and stimulant effect, to wrap the electrode with absorbent cotton and apply iodized phenol, U. S. P., with a current of ten to fifteen milliamperes for a few minutes for its cataphoretic effect. In the dysmenorrhœa and leucorrhœa resulting from virginal endometritis we should supplement the usual hygienic and medicinal regulation of the pelvic functions with the stimulant applications of the galvanic current by means of large electrodes to the back and hypogastrium, thereby assisting the natural defenses to repel what is perhaps a constant invasion of the uterine cavity. Local examinations in these cases are not warrantable until these measures have been given a thorough trial.

### POST-PUERPERAL METRITIS.

There is perhaps no remedy so effectual in this trouble, whether due to inertia or infection, as electricity. If a case of simple inertia two or three applications of the primary faradic current will be sufficient to tone up the muscular fibers, and rapid involution is the result. If caused by shreds or small particles of retained placenta, abnormal conditions of the endometrium or traumatic accidents, the galvanic current is indicated. In the former the positive pole by greatly increasing drainage is sufficient to bring away the small particles of retained placenta and shreds of membranes, but if any considerable quantity of these be retained the application should be preceded by the curette. When hemorrhage is present, the carbon electrode is preferable. If not, the covered elastic electrode should be inserted thoroughly up to the fundus and then slightly withdrawn to prevent the electrolytic action on the fundus. A dosage of from twenty to fifty or sixty milliamperes should be used alternately, increasing and diminishing the current strength. This followed by the faradic current. A ready response may be expected from this treatment.

ENGORGEMENT.

Here we have not only increased size and weight of the uterus, but stasis or congestion. As the disease progresses, chronic stagnation. For recent cases of engorgement the production of local anæmia would seem to be indicated, but for the chronic form the repeated production of temporary hyperæmia by means of negative primary faradism with the intra-cervical electrode. The positive being placed on the back or lower abdominal region has brought the best results. Faradization here produces a decided but temporary hyperæmia of the uterus, and by its effect on the disturbed circulation furnishes a means of drainage and possible absorption of existing exudates. The use of the bipolar intra-uterine electrode is also highly commended in this condition by Apostoli and others, while men of equal eminence sustain the monopolor method. Applications should not last over five to eight minutes, and, as soon as contractions are readily induced, three minutes should be the average duration.

AMENORRHOEA AND SCANTY MENSTRUATION.

Jenks, Beard and Rockwell, Munde and Smith cite many cases of amenorrhœa, or scanty menstruation, in young girls, due either to the intensity or overwork in the pursuit of education or accomplishments, or to constipation. In undergoing the mental strain usually necessary in preparing for school examinations and in the pursuit of literary attainments the brain uses up the good rich blood that the generative organs need so much just at this time of development. Again, constipation, on account of its frequency in young girls and its relation to the subject in hand, deserves special mention. Its effect of producing a chain of nervous phenomena as long as an unwritten code of ethics is well known. Perhaps the most serious, here, is the disgust for food and its resultant anæmia. I have recently treated a case of this character with a history of constipation of two years standing. Condition from beginning gradually increasing in severity until at the end of twenty months bowels only moved about once in fourteen or fifteen days, when she applied for treatment. Was decidedly anæmic, tongue furred, breath offensive, and amenorrhœa the trouble for which relief was sought. A decided aversion for food was elicited upon inquiry.

We find anæmia and its corresponding decrease in vitality from any cause frequently with its symptom of amenorrhœa. In another class we have possibly only a local anæmia. I refer to an indolent class of women who, especially after marriage, developed obesity. These cases are not only relieved by the secondary negative, but the obesity seems to be checked by the treatment. In the first class of these cases electricity is of little value except for its general effect of stimulation. In the cases caused by constipation I believe it to be the most effective remedy at our disposal. With one electrode on the spine, and the other, a roller electrode, properly applied to the abdomen, and the effect a general faradization of the bowel, we relieve the constipation and at the same time stimulate the nervous and muscular system of these organs to the proper performance of their respective functions.

DYSMENORRHOEA.

Smith, in Bigelow's International System of Electro-therapeutics, classifies the various forms of this distressing condition as neuralgic, congestive, obstructive, membranous and ovarian. The neuralgic form is due to either an inferior quality or insufficient quantity of circulatory nourishment. Faradism by increasing the activity of the uterine muscles and improving the circulation is a valuable adjunct to other treatment. The congestive form has been considered under the head of engorgement. In the constructive form we may have, pathologically, a flexion, and organic or spasmodic stricture or a polypus or tumor filling up the cervical canal. The negative pole of the galvanic current offers advantages in the obstructive form not to be had from any other agent. On even small milliamperage it has the power to dissolve or dilate the stricture effectually. Applications made with a uterine probe (and for this purpose I have used the ordinary flexible block tin electrode with flexible insulator) will generally be followed by the desired results. The greatest care is necessary in making the applications that no mechanical injury from undue pressure may result. In passing the electrode when obstruction is met, very slight pressure should be made, and after three to five minutes the sound glides by or through the obstruction without effort on the part of the operator. The application should be repeated every five to seven days, selecting a larger sound at each seance until the desired degree of dilatation be procured. In obstruction from polypus I have found no experience, but it is claimed that the electrolytic effect of the current brings about disorganization and consequent discharge of the tumor. In the membranous form a dosage of from fifty to one hundred milliamperes is necessary, according to Smith, of Montreal. He secures the disorganization of the thickened endometrium, the dilatation of the internal os, producing better drainage and the not well defined interpolar action on the trophic nerves hastening the process of fatty degeneration and consequent softening. Tubal dysmenorrhœa is due to inflammatory deposits and cicatrices binding down the tube and preventing the free peristalsis of same, hence the pain. Absorption through means of the galvanic current and liberation of the tube in many cases may be had.

MENORRHAGIA.

As a primary cause of menorrhagia we often find an obstruction to the venous circulation. Signified by a thickened endometrium, almost filled up with blood-vessels whose walls are so weak that they rupture on the slightest provocation. The most careful introduction of a sound is frequently followed by profuse bleeding. Prominent among the causes for this condition are those of uterine enlargement with mal-positions, fungous endometritis, fibroids, cervical lacerations and cancer. If the two poles of a galvanic current be placed in a saline solution, the solution will be decomposed, acids coming to the positive and the base to the negative. The same chemical decomposition in induced when the poles are placed in the saline tissues of the body, hence we get the acid or alkaline caustic effects at the point of treatment, according to the pole used. The intrauterine positive, when used in sufficient strength to cauterize, say fifty to one hundred milliamperes, will bring about an exfoliation of the spongy and vascular mucous membrane and at the same time acting as a sure and safe hæmostatic, reducing the size and weight of the organ, and by its interpolar action strengthening the uterine supports. According as these effects are produced, we find an equalized circulation and relief.

Before closing, it is my desire to say a few words about peri- and parametritis and post-inflammatory deposits, conditions that the physician must meet and contend with not infrequently.

Electricity in peri- and parametritis is ordinarily confined to the subacute and chronic stages, but Apostoli strongly advocates the use of the current of tension by means of the bipolar vaginal electrode in the acute stage. We find others of ripe experience with this agent heartily agreeing with him. In the subacute stage, or as soon as the sound can be introduced into the uterus without pain, the bipolar uterine electrode may be used with the same current until the condition will tolerate small and gradually increasing currents of positive galvanism as an adjunct to the above treatment. Goelet, in Section G, page 127, of International System of Electro-therapeutics, prefers the vaginal ball electrode for the galvanic treatment and the continuation of the vaginal bipolar faradism. Where the exudation is recent, rapid absorption usually follows these procedures. If the mass is adherent or closely connected with the uterus, or a metritis or endometritis co-exist, intra-uterine galvanism should be begun as soon as tolerance is brought about by the other current. Great caution should be observed in using electricity in these troubles. The faradic current of tension never being pushed farther than the point of comfortable tolerance, and may be continued until distinct relief from pain (in the acute state of the disease) is brought about. Usually fifteen to twenty minutes. When galvanism is begun, only thirty to fifty milliamperes should be used for three minutes, with intervals of four to seven days. The dosage gradually increased as tolerance is established. In obstinate exudations situated near the vaginal walls galvano-puncture is indicated. First the positive, because it is less likely to create irritation and is often followed by complete resolution. In case of failure after a sufficient number of these applications are made then the negative is resorted to. Such applications should invariably be preceded by an antiseptic douche, and followed by the same, with the addition of a loose tampon of iodoform or creolin gauze placed against the site of puncture.

In closing, I desire to say that I would not have it understood that I exclude other appropriate treatment while using electricity, but have simply, as nearly as possible, confined my remarks to the subject in hand. In this paper I have been unable to do more than merely outline a few of the important subjects, not even touching some of the gynæcological diseases which are perhaps more amenable to this agent than those gone over.

